# Risk of *Trypanosoma cruzi* infection among travellers visiting friends and relatives to continental Latin America

**DOI:** 10.1371/journal.pntd.0009528

**Published:** 2021-07-02

**Authors:** Adrián Sánchez-Montalvá, Catalina Salinas, Elena Sullerio, Fernando Salvador, Pau Bosch-Nicolau, Clara Crespillo-Andújar, Elena Trigo, Sílvia Roure, Lluís Valerio, Juan Espinosa-Pereiro, Israel Molina

**Affiliations:** 1 Infectious Diseases Department, Vall d’Hebron University Hospital, Universidad Autónoma de Barcelona, PROSICS Barcelona, Barcelona, Spain; 2 Zoonoses and One Health Masters Program. Universitat Autònoma de Barcelona, Barcelona, Spain; 3 Microbiology Department, Vall d’Hebron University Hospital, PROSICS Barcelona, Barcelona, Spain; 4 National Referral Centre for Tropical Diseases, Infectious Diseases Department, Hospital Universitario Ramón y Cajal, Instituto Ramón y Cajal de Investigación Sanitaria, Madrid, Spain; 5 Travel and Tropical Medicine Referral Unit. La Paz-Carlos III University Hospital, Madrid, Spain; 6 North Metropolitan International Health Unit, PROSICS (International Health Program of the Catalan Health Institute), Universitat Autònoma de Barcelona, Barcelona, Spain; Federal University of Ceará, Fortaleza, Brazil, BRAZIL

## Abstract

**Background:**

Chagas disease (CD) is regarded as a possible risk for travellers to endemic areas of continental Latin America (LA). The aim of the study is to determine the risk of *Trypanosoma cruzi* (TC) infection among travellers to CD endemic areas and to identify risk factors for acquiring TC infection.

**Methods/Principal finding:**

We designed a multicenter cross-sectional study among travellers in Spain (Badalona, Barcelona and Madrid). All available adults with laboratory confirmed proof of absence of TC infection from January 2012 to December 2015 were contacted. Participants referring a trip to LA after the negative TC screening were offered to participate. We performed a standardized questionnaire of travel related factors and measurement of TC antibodies in serum. A total of 971 participants with baseline negative TC serology were selected from the microbiology records. After excluding participants not meeting inclusion criteria, eighty participants were selected. Sixty three (78.8%) were female, and the median age was 38 (IQR 34–47) years. The reason to travel was visiting friends and relatives in 98.8% of the participants. The median duration of travel was 40 (IQR 30–60) days, with 4911 participants-day of exposure. Seventy seven cases (96.25%) participants had two negative TC serology tests after the travel, two cases (2.5%) had discordant serology results (considered false positive results) and one case was infected before travelling to LA. According to our data, the upper limit of the 95% confidence interval of the incidence rate of TC acquisition in travellers is 0.8 per 1000 participant-days.

**Conclusions/Significance:**

Among 79 non-CD travellers to TC endemic areas, we found no cases of newly acquired TC infection. The incidence rate of TC acquisition in travellers to endemic countries is less than or equal to 0.8 per 1000 traveller-days.

## Author summary

Chagas disease is caused by the protozoan parasite *T*. *cruzi*. It is endemic in certain areas of continental Latin America. Few cases of *T*. *cruzi* infection have been described in travellers. However, there is little information regarding the incidence rate of T. cruzi infection during a trip to continental Latin America. In this study we aim to study the incidence of T. cruzi infection among migrants from Latin America living in Spain travelling to visit friends and relatives. In this study we found no cases of newly acquired *T*. *cruzi* infection among 79 previously uninfected travellers and calculate that the upper limit of the 95% CI of the incidence rate of *T*. *cruzi* acquisition in travellers is 0.8 per 1000 participants-day.

## Introduction

Concerns that travel to Chagas disease (CD) endemic areas of continental Latin America may pose a risk to acquire CD have been proposed and are part of health travel recommendations. The Yellow Book from Centers for Disease Control and Prevention (CDC) states that “the risk to travellers is extremely low, but they could be at risk if staying in poor-quality housing or from consuming contaminated food or beverages in endemic areas.”[1] Global mobility and travel patterns are rapidly evolving, modifying the dynamic of endemic diseases. CD, once restricted to continental Latin America, is also prevalent in other countries with migrants from this area. Outside Latin America, United State of America and Spain have the largest population with CD. [1] The reason to travel is usually associated with the risk of travel-related diseases. Travellers visiting friends or relatives (VFR) are a group with increased risk of travel-related diseases. Despite there are different definitions for VFR, it usually refers to travellers whose primary purpose of travel is to visit friends or relatives and for whom there is a different epidemiologic risk of acquiring a diseases between the origin and the destination. The great majority of VFR travellers are individuals born abroad returning to their home countries to visit family and friends, although other individuals with other circumstances are also included. [1] In 2010, the European Travel Network reported 60 cases of chronic CD among 7408 returning travellers across Europe.[2] It is possible that these cases of chronic CD reported among VFR travellers actually represent chronic infections not detected prior to travel since pre-travel CD serologies is not routinely performed.

The causal agent of CD, or American trypanosomiasis, is the protozoan parasite *Trypanosoma cruzi*. CD is endemic in 21 countries in continental Latin America and is most commonly transmitted through triatomine vectors, which are obligate blood-feeder insects. Other routes of transmission are also possible including mother-to-child transmission, oral transmission through beverages infected with the *T*. *cruzi* and through blood transfusion and organ transplantation. *T*. *cruzi* life cycle encompasses a complex interaction between the wild and domestic environment, where multiple actors are involved. Human activities in enzootic areas modify the behaviour of the vector and wild animal increasing the likelihood of human-vector interaction.[3] Triatomine infestation of human dwellings is facilitated by certain construction materials: cracks in mud or concrete walls, junctions between adobe bricks, wooden or cane walls, roofs made of palm trees, and earthen floors have been implicated.[4] Having poultry indoors has also been associated with the presence of triatomine in human dwellings.[5] Fully stuccoed-walls have been identified as a protective factor for both human dwellings and animal enclosures.[6]

As vector control and surveillance system improve other transmission routes have become more evident. Oral transmission of *T*. *cruzi* may lead to acute cardiac alterations from massive parasitemia.[6] Isolated cases and outbreaks have been reported in Argentina, Brazil, Colombia, and Ecuador.[7–9] Reported food vehicles of *T*. *cruzi* infection include sugar cane, açaí juice, palm wine, and wild game.[10] The interaction between humans and triatomine vectors has determined transmission in endemic areas, but globalization is now determining epidemiology trends throughout the world.

Transmission related to blood products, donation of organs, and mother-to-child occurs worldwide with serious public health implications. [10] For non-endemic countries, determining the risk of acquiring Chagas disease after travel to endemic areas would contribute the evidence necessary to support pre-travel advice at the clinical level, and screening efforts at the policy-maker level. Risk of acquiring CD has been deemed to be minimal predicated on cases reported, although risk estimation in low and high risk travellers is lacking. The main objectives of this study were 1) to determine the frequency of *T*. *cruzi* infection acquired during the travel among returning VFR travellers to *T*. *cruzi* endemic areas, 2) to describe VFR travellers demographic and travel characteristics, and 3) to identify possible risk factors for acquiring *T*. *cruzi* infection.

## Methods

### Ethics statement

The Vall d’Hebron institutional review board provided ethical clearance (local review board code number: PR(AG)193/2017). Patients were asked for an oral consent at first telephone contact. Written consent was obtained for all patients that agreed to participate in the study.

### Study design

This is a multicenter cross-sectional study performed from January to December 2017. We recruited all adult subjects with two negative *T*. *cruzi* serology tests from January 2012 to December 2015 as provided by the Microbiology Department at 3 Spanish International Health Units: Vall d’Hebron University Hospital (Barcelona, Spain), Hospital La Paz-Carlos III (Madrid, Spain) and Metropolitana Nord Clinic (Badalona, Spain). Travel history was elicited by telephone after a brief description of our research and the verbal consent of the patient was granted orally. Eligible subjects were those who travelled to continental Latin America at some point after their latest negative *T*. *cruzi* testing. Participants with a recent travel history were requested to wait 2 months before venipuncture. Three telephone calls were made before determining a participant as non reachable. Reachable participants were invited to participate and visit the International Health Unit. Reminders via telephone and text message were sent to ensure attendance. All participants provided a written informed consent, a standardized questionnaire, and a blood sample for *T*. *cruzi* serology testing. Exclusion criteria were: previous positive or discordant *T*. *cruzi* serology, negative serology after successful treatment for Chagas disease, no history of travel to CD endemic area, participants younger than 18 years and deceased subjects.

We analyzed demographic and travel characteristics of participants, determined the frequency of newly acquired *T*. *cruzi* infection, and identified risk factors of *T*. *cruzi* infection during travel. Consumption of fresh fruit juices and sugar cane was grouped as “beverages”. Accommodation built of adobe, palm tree roofs, walls of cane, or walls of wood was grouped as “house construction materials” for analysis. “Reason for travel” was categorized into tourism, business, research or education, missionary or volunteer, and VFR, according to GeoSentinel classification.[11]

The diagnosis of CD was established by the detection of *T*. *cruzi* antibodies using two different techniques as recommended by the World Health Organization (WHO).[12] Interpretation of the techniques was performed according with the manufacturer’s instructions. Final outcome was considered as follow: positive results to two serologic techniques were considered diagnostic of *T*. *cruzi* infection, negative results to two serologic techniques ruled out *T*. *cruzi* infection, and one positive and one negative result was considered discordant, and further investigations were recommended. Vall d’Hebron University Hospital and Metropolitana Nord Clinic used two enzyme-linked immunosorbent assays (ELISA), one with recombinant antigen (Bioelisa Chagas, Biokit, Lliçà d’Amunt, Spain) and the other with crude antigen (Ortho *T*. *cruzi* ELISA, Johnson and Johnson, High Wycombe, United Kingdom). Hospital La Paz-Carlos III used one ELISA (Vircell Microbiologist, Spain) and one IFI (Indirect Immunofluorescence assay; IFA test system, Trinity Biotech, Ireland). A rapid immunochromatographic test (ICT; SD BIOLINE Chagas Ab Rapid, Standard Diagnostics, INC, Republic of Korea) that detects the colorimetric antigen-antibody reaction on a membrane is also available. An Internal Board encompassed by 2 clinicians and 2 microbiologists reviewed any positive serology testing for interpretation of the final outcome.

### Statistical analysis

The sample size was a convenience sample, determined by all available eligible subjects between 2012 and 2015, and their willingness to participate. Data were analyzed with STATA (v.14) software. Median and interquartile range (IQR) were calculated for quantitative variables, and frequencies and percentages were calculated for qualitative variables. A regression model was planned to identify risk factors among those with *T*. *cruzi* infection and self-reporting triatomine bugs observation. Variables with a p-value≤0.001 in the univariate analysis were included in the multivariable logistic regression model. The analysis to identify risk factors among infected participants could not be performed as none of the participant developed the infection during the study period. Incidence rate of *T*. *cruzi* acquisition was calculated as the number new cases divided by total participant-days. 95% upper limit of incidence rate was calculated based on Poisson distribution using Epidat software version 3.1.

## Results

**Fig 1:**
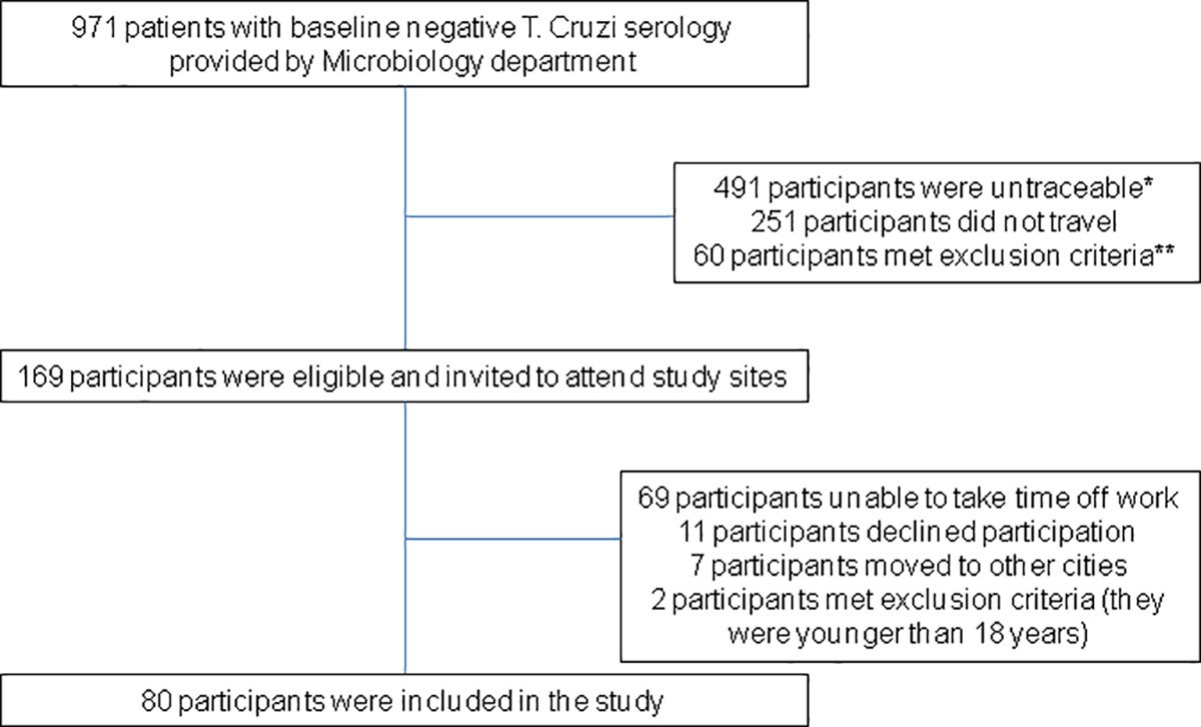
Participants flow diagram. * Participants were untreaceable due to: 308 no one answered the telephone, 86 telephone number did not exist, 26 wrong telephone number, 7 restricted in-coming calls, 20 had no telephone number in the medical record, 15 returned to home countries. **Participants meeting exclusion criteria: 3 had discordant serology tests, 5 had seroreversion after Chagas’ disease treatment, 22 were deceased and 28 were younger than age 18.

Of the 971 subjects with baseline negative *T*. *cruzi* serology between January 2012 and December 2015, 169 (17.4%) participants were eligible for study participation. Eighty participants visited the International Health Units and were included in the study and analysis. See **Fig 1** for patient flow diagram.

Median age was 38 years (IQR 34–47) and 63 (78.8%) were female. The reason to travel was VFR in 98.8% of the participants. The median duration of travel was 40 days (IQR 30–60). Country of birth and country of travel were the same in all but 2 participants: one patient from Bolivia visited relatives in Argentina, and one patient from Colombia visited Peru. Ninety percent of the participants travelled for at least 3 weeks. Rural areas were visited by 63.8% of the participants and more than 50% were in contact with farm animal, mainly poultry, swine and cattle. Given the exploratory nature of this study, the participants were interviewed with open questions like exposure to peridomestic farm animals during travel. More data are shown in **Table 1**.

**Table 1:** Demographic and travel characteristics of study population (n = 80). VSF: visiting friends and relatives.

Descriptive data	Frequency^a^	%^b^
**Demographic characteristics**		
Female sex	63	78.8
Median age (years)	38	IQR 34–47
**Travel characteristics**		
Travel destination		
Bolivia	32	40
Ecuador	14	17.5
Perú	9	11.25
Colombia	7	8.8
Honduras	6	7.5
Brazil	4	5
Argentina	3	3.8
Paraguay	3	3.8
Nicaragua	1	1.3
Venezuela	1	1.3
Reason for travel		
VFR	79	98.8
Business	1	1.2
Duration of travel (days)		
1–30	33	41.3
31–60	28	35
61–90	11	13.8
>90	8	10
Visited rural areas	51	63.8
Peridomestic farm animals	42	52.5
Dogs in or around house	58	72.5
Cats in or around house	42	52.5
Fever during travel	14	17.5

^a^ Absolute frequency for all variables except “Age” which reflects the median value

^b^ Percentage for all variables except for “Age” which reflects interquartile range

**Table 2** shows exposure to risk factors for *T*. *cruzi* infection. A total of 71.3% of the participants consumed any beverages associated with CD oral infection. Regarding the building materials of accommodation during travel, 17 (21.3%) participants reported staying at a house built of *adobe;* 3 (3.8%) reported palm tree roof; 4 (5%) reported wooden walls; and 2 (2.5%) reported cane walls. Accommodation built by any of these materials was reported by 18 (22.5%) of participants. Self-reported insect bites were referred by 55% of the participants; none of them were attributed to triatomine bugs. Surprisingly, 66.3% of the participants reported on the observation of triatomine bugs. No participant referred blood transfusion, hospitalization or surgical procedures during the trip.

**Table 2:** Exposure to well described risk factors for *T*. *cruzi* infection (n = 80).

Risk factors described in the literature	Frequency	%
Beverages^a^ Fresh fruit juices only Sugar cane only Both	57 26 4 27	71.3 32.5 5 33.8
House construction materials^b^	18	22.5
Self-reported any insect bite	44	55
Observation of Reduviid bugs	53	66.3
Self-reported Reduviid bug-bite	0	0
Blood transfusion	0	0

^a^ Refers to exposure to implicated beverages (fresh fruit juices and/or sugar cane).

^b^ Refers to exposure to accommodation built of adobe, roof made of palm trees, walls of wood, and/or walls of cane.

Risk factors associated with self-reporting triatomine bug observation in VFR returning travellers in the univariate analysis were returning from Bolivia, consumption of implicated beverages and having acquaintances with CD. After the multivariable analysis, only the consumption of beverages remained statistically significant. More information is shown in **Table 3.**

**Table 3:** Factors associated with self-reporting triatomine bugs observation.

	Triatomine bugs sighting (n = 53)	No triatomine bug sighting (n = 27)	p value, univariate analysis	OR (95% CI), univarate analysis	Adjusted OR (95% CI)
Sex, female	40 (75.5%)	23 (85.2%)	0.31	0.5 (0.2–1.8)	
Age, <35 years	8 (29.6%)	22 (41.5%)	0.3	0.6 (0.2–17)	
Visiting rural areas	34 (64.2%)	17 (63%)	0.92	1.1 (0.4–2.9)	
Destination, Bolivia	28 (52.8%)	5 (18.5%)	**0.003**	**4.8 (1.6–14.5)**	2.1 (0.6–8.2)
Contact with farm animals	28 (52.8%)	14 (51.9%)	0.93	1.1 (0.4–28)	
Contact with dogs	41 (77.4%)	17 (63%)	0.17	2 (0.7–5)	
Contact with cats	32 (60.4%)	10 (37%)	0.048	2.7 (1–7.1)	
Consumption of implicated beverages	43 (81.1%)	14 (51.9%)	**0.006**	**4.4 (1.6–12.6)**	**3.3 (1.1–9.8)**
Relative or friend with CD	28 (52.8%)	4 (14.8%)	**0.001**	**6.7 (2.0–22.1)**	3.8 (0.9–15.8)
House construction materials^a^	13 (24.5%)	5 (18.5%)	0.54	1.5 (0.5–4.7)	

Note: CD, Chagas disease

^a^ Refers to exposure to accommodation built of adobe, roof made of palm trees, walls of wood, and/or walls of cane.

Among our group of returning VFR travellers to endemic areas, 79 out of 80 (98.75%) had a non positive *T*. *cruzi* serology after travel, being negative in 77 participants (96.25%) and discordant inconclusive serology (probably false positive results) in 2 participants. One patient was considered to have had *T*. *cruzi* infection before the trip according to the trace-back efforts that included medical records and blood transfusion infection diseases screening records. **Table 4** shows the trace-back investigation of the discordant and positive case, along with the internal board final decision. Therefore, according to our data, the upper limit of the 95% confidence interval of the incidence rate of *T*. *cruzi* acquisition in travellers is 0.8 per 1000 participant-days.

**Table 4:** Trace back investigation of participant with a positive serology testing and internal board final decision (shade results are before traveling to *T*. *cruzi* endemic countries).

Case 1 (Bolivia, Cochabamba)								
Transfusion center								
	Dec 2013	May 2014		Aug 2014	Jan 2015		Jul 2016	Jan 2018
ELISA (native)	Inderterminate	Negative		Negative	Negative		-	-
IFI	Indeterminate	Indeterminate		Indeterminate	Positive		-	-
ELISA (recombinant)	Positive	Positive		Positive	Indeterminate		-	-
PCR peripheral blood	-	Negative		Negative	-		-	-
Tertiary Hospital								
Rapid Test (ICT)	-	Negative		Negative	-		Negative	Negative
ELISA	-	Negative		Negative	-		Negative	Indeterminate
IFI	-	-		-	-		-	positive
PCR peripheral blood	-	-		-	-		-	Negative
Internal Board Final Decision					Inconclusive			
Case 2 (Bolivia, Cochabamba)								
Transfusion center								
	Jul 2016		Jan 2018			Feb 2018		
ELISA (native)	-		-			-		
IFI	Positive		-			-		
ELISA (recombinant)	-		-			-		
Chemiluminescence	Positive							
PCR peripheral blood	-		-			-		
Tertiary Hospital								
Rapid Test (ICT)	Negative		Negative			Negative		
ELISA	Negative		Positive			Positive		
IFI	-		Positive			Positive		
PCR peripheral blood	Negative		Negative			Negative		
Internal Board Final Decision			Chronic infection previous to travel (2016 Hospital False negative)					
Case 3 (Argentina, Buenos Aires)								
Tertiary Hospital								
	Dec 2015		May 2017			Aug 2017		
ELISA (recombinant)	Negative		Positive			Positive		
ELISA (native)	Negative		Negative			Negative		
PCR peripheral blood	-		-			Negative		
Internal Board Final Decision			Inconclusive					

*ELISA*: enzyme-linked immunosorbent assays*; ICT*: *immunochromatographic serological test; IFI*: *indirect immunofluorescence; PCR*: *Polymerase chain reaction;*

## Discussion

Our study assesses the risk of acquiring CD after travelling to continental Latin America exclusively among participants documented to be free of *T*. *cruzi* infection before travel. Other publications have reported cases of *T*. *cruzi* infection among travellers.[2] However, no documentation of *T*. *cruzi* infection-status before travel was available, precluding us from assuring that those cases were infected during travel to endemic areas. Our study participants had a baseline *T*. *cruzi* testing as part of screening programs of asymptomatic migrants of Latin American origin at the Infectious Diseases Department, routine blood bank screening protocol, congenital transmission prevention program, diagnostic workup of clinical findings compatible with CD, prior to immunosuppressant therapy, biologic therapy, or organ transplantation. Among 79 adult VFR travellers to continental Latin America and who were free of CD prior to travel, we found no cases of *T*. *cruzi* infection and found 2 patients with discordant serology that were classified as inconclusive and probably false positive results by our Internal Board. In our study, the incidence rate of acquisition of *T*. *cruzi* infection in travellers to Latin America CD endemic areas is less than is 0.8 per 1000 participants-day.

VFR travellers in this study were migrants from Latin America living in Spain with demographic characteristics similar to Latin American migration to Spain. However, the methodology of the study may have introduced a selection bias, limiting the extrapolation of the data to a broader population. Similar to a previous European studies, a female predominance among Latin American VFR travellers was noted.**[]** [13,14] The median duration of travel was 40 days (IQR 30–60 days), which is much longer than that found in other series of ill returning travellers (median 17 days, IQR 8–43).[15] Exposure to risk factors for *T*. *cruzi* infection described in the literature was assessed with a standardized questionnaire. Fresh fruit juices and sugar cane beverages were common practices among our study cohort. These beverages can be contaminated with the *T*. *cruzi* infected vector itself or its faeces and be inadvertently ingested. [7–9] Regarding the quality of accommodations, 22.5% of participants stayed at a house built of adobe, palm tree-thatching, wooden walls, or cane walls. Poor dwelling conditions are associated with triatomine bug domestic infestation, increasing the risk of *T*. *cruzi* infection acquisition. Despite efforts aimed at vector control have been successfully implemented in Latin America since the 1990s, with a significant reduction of vectorial transmission in many countries and eradication in others, it seems necessary to maintain such programs and implement a permanent surveillance system to rapidly detect good dwelling practices deterioration or/and triatomine infestation.[4]

We found 3 cases with at least one positive serology test result. After carefully evaluation of the trace back investigation results by the Internal Board, 1 case was considered to be infected before the trip and the other 2 cases were deemed as inconclusive (probably false positive results). Discordant serology testing poses a diagnostic challenge for the clinician. A study by Moure et al using a western blot technique (TESA-Blot (Biomérieux, RJ, Brazil)) was evaluated as a confirmatory diagnostic tool in patients with inconclusive and discordant serology helped establish the diagnosis of CD in half of cases, unfortunately the technique was not available in our laboratories for further investigation.[16] These patients will be followed up in clinic with repeated serology every 6 months.

Currently, clinical trials of new treatment in CD include travelling to endemic areas as exclusion criteria limiting the number of available participants or withdrawing participants due to unexpected trips.[17,18] According to our results this exclusion criteria should be reconsidered in future clinical trials, as risk of *T*. *cruzi* reinfection is minimal. However, trials developed in areas where vectorial transmission is still present should try to minimize the risk of reinfection by implementing strategies to improve dwelling conditions, to undertake peridomestic conditioning, to induce behavioural modifications, to work in the relocation of animal facilities and further work into vector control strategies. [19,20]

This study has several limitations. First, we selected subjects that had already approached the health care system, and tested negative for CD, so this strategy might have pre-selected subjects with a lower predisposition or from areas with lower incidence of CD. However, many of them had relative or friends with CD, which may indicate current or previous active transmission in the visit area. Second, the necessity to reach participants by telephone to assess for eligibility significantly reduced our sample size since almost half of the subjects from our database were untraceable after several attempts. This was especially true for the years 2012 and 2013. Presumably these subjects do not keep the same telephone number for a long period of time. Another limitation was that half of eligible subjects were unable to come to the study site due to schedule or distance limitations. Jobs filled by foreign workers often have longer hours and offer little or no time flexibility. Female participants tended to work caring for children or the elderly, which gave them more time flexibility than their male counterparts. Participants may have wanted to “save” work leaves for more urgent needs. Working hours at our study site overlapped with usual working hours, which may have introduced a selection bias to represent more females.

## Conclusions

Among 79 non-CD VFR travellers to continental Latin America, we found no cases of newly *T*. *cruzi* infection. Two participants had inconclusive results due to discordant results, likely from false positive results. The incidence rate (risk of acquiring) of *T*. *cruzi* acquisition in travellers to endemic countries is less than or equal to 0.8 per 1000 travellers-day. This study may help improve pre-travel health counselling and better inform travellers to continental Latin America of the low risk of *T*. *cruzi* infection acquisition. However, mitigating action should still be recommended. Besides, they can also serve to minimize the risk of acquiring other travel-related infections.
